# Male Sexual Dysfunction and Chronic Kidney Disease

**DOI:** 10.3389/fmed.2017.00032

**Published:** 2017-03-22

**Authors:** Matthew M. Edey

**Affiliations:** ^1^Department of Nephrology, Hull and East Yorkshire Hospitals NHS Trust, Kingston upon Hull, UK; ^2^Hull-York Medical School, Kingston upon Hull, UK

**Keywords:** chronic kidney disease, hypogonadism, erectile dysfunction, sexual dysfunction, testosterone

## Abstract

Male sexual dysfunction is common in chronic kidney disease (CKD), particularly in end-stage renal disease. Historically, this cause of considerable morbidity has been under-reported and under-recognized. The ideal approach to diagnosis and management remains unclear due to a paucity of good quality data, but an understanding of the pathophysiology is necessary in order to address the burden of this important complication of CKD. This paper will review the endocrine dysfunction that occurs in renal disease, particularly the hypothalamic–pituitary–gonadal axis, discuss the causes of erectile dysfunction, infertility, and altered body image and libido in these patients and suggest appropriate treatment interventions.

## Sex Hormone Abnormalities in Chronic Kidney Disease (CKD)

No organ system functions or fails in isolation. As CKD progresses, so the accumulating physiological derangement disturbs the activities of other organs and tissues. With CKD, many components of the endocrine system are influenced. This is most evident in the extreme case, dialysis-treated end-stage renal disease (CKD stage 5-D). Disruption to the hypothalamic–pituitary–gonadal axis in men with CKD results in significant functional impairment and quality of life is affected.

A reduction in renal function may affect hormonal actions in a number of ways. Many hormones are metabolized or excreted by the kidneys. Sensitive feedback mechanisms are destabilized by CKD. Circulating binding proteins can alter in concentration, where there is renal dysfunction. Hyperprolactinemia and hypogonadism are two significant endocrine disturbances that occur in the setting of CKD.

### Pituitary and Hypothalamic Alterations

In the normal state, pulsatile hypothalamic secretion of gonadotropin-releasing hormone (GnRH) stimulates the production of luteinizing hormone (LH) and follicle-stimulating hormone (FSH) by the pituitary gland. In turn, pituitary gonadotropin (LH and FSH) secretion is also pulsatile. LH is the prime regulator of testosterone production. FSH, in concert with high tissue testosterone levels, stimulates spermatogenesis.

In CKD, pituitary LH secretion maintains its pulsatile character, but the amplitude of the pulses is diminished (1–3). The baseline levels of LH are high, due in part to feedback from low testosterone levels and in part to reduced renal clearance. In addition to these secretory changes, the normal signaling of luteinizing hormone is inhibited in CKD (4). This appears to occur in a proportionate degree of severity to the reduction in glomerular filtration rate (GFR).

The explanation for this aberrant gonadotropin secretion may lie within the hypothalamus or the pituitary. Defects in both hypothalamic function and pituitary gonadotropin release have been postulated, with some supporting evidence in CKD (3). The administration of clomiphene induces an appropriate increase in LH and FSH in men with CKD, suggesting that a hypothalamic derangement is “holding back” normal pulsatile GnRH secretion. Similarly, GnRH infusion augments LH production in subjects with CKD, as would be expected in normal individuals. However, the same blunted waveform of pulsatile LH secretion described above persists, which points to a pituitary problem. Despite this, the exact mechanisms for these endocrine abnormalities remain obscure.

### Changes in Testosterone Production

Testosterone deficiency (or hypogonadism) is common in patients with CKD and particularly in those receiving dialysis. Published data suggest perhaps 40–60% of hemodialysis patients exhibit hypogonadism (5, 6), and a lower figure of around 15–40% applies to CKD stages 1–4 (7). This prevalence is notably in excess of the general population rate. Insufficient gonadotropin stimulation contributes to the etiology, but other factors are also significant. The production of testosterone naturally declines with age, and there is increased circulating sex hormone-binding globulin (SHBG) which in turn reduces free testosterone. The increasing age of the dialysis population means that the age-related fall in testosterone activity serves to exacerbate the effects of CKD.

Primary hypogonadism has been implicated by studies showing a blunted testosterone response to human chorionic gonadotropin stimulation (8, 9). Testicular size is reduced in CKD and histological abnormalities are identifiable, including seminiferous tubular derangement, interstitial fibrosis, and areas of calcification.

Serum testosterone levels have been shown to predict mortality in hemodialysis patients, although increasing age is a confounding variable and appears to account for much of the elevated mortality risk (6).

### Changes in Prolactin Secretion

Prolactin is secreted principally by the anterior pituitary gland, with a contribution from peripheral tissues (10). Its function in women is to control breast development and lactation but in men its actions are less well understood. Hyperprolactinemia occurs commonly in CKD, with a prevalence of 30–65% (11–13). It is understood that this excess is due to a combination of diminished prolactin removal by the kidney and increased synthesis (14). The concept of renal clearance of prolactin in the physiologic state is not universally accepted, but a single and somewhat venerable study has shown a reduced metabolic clearance of prolactin in CKD stage 5-D (15), and an earlier publication reported a 16% reduction in prolactin concentration between renal artery and vein (12). The consequences of hyperprolactinemia are most marked in patients receiving dialysis therapy. The endocrine complications of hyperprolactinemia are manifest through disruption of pulsatile GnRH. This leads in turn to hypogonadotropic hypogonadism. The clinical features may include erectile dysfunction (ED), decreased libido, infertility, gynecomastia, and reduced skeletal mass (10, 16). It has been shown that elevated circulating prolactin levels are associated with cardiovascular events in men with ED (17). This has been reproduced in a study of CKD patients, incorporating two independent cohorts of hemodialysis and CKD stage 1 to CKD stage 5-ND subjects (14).

### SHBG and Other Associated Hormonal Alterations

Sex hormone-binding globulin is the principal binding protein for testosterone in the circulation, with approximately 70% of the circulating hormone bound to it in normal circumstances. It is usual in men for SHBG levels to rise with advancing years, thus reducing available testosterone and exacerbating the age-related decline in testosterone production. In CKD, it appears that SHBG levels are unaffected by a decline in GFR. When adjusted for confounding factors, subjects with normal renal function or CKD stages 3–5-ND have similar measured SHBG (18). In a study of men with type 1 diabetes, progressive renal disease was not associated with SHBG alterations (19). It has been shown that a change from standard hemodialysis treatment for men with CKD stage 5-D to alternate nightly, longer sessions results in increased testosterone levels, while measured SHBG is unaffected (20). This suggests that greater delivery of dialysis improves testosterone bioavailability but not through SHBG effects.

### Comorbidities in CKD Patients That Affect Sex Hormones

Those with CKD typically have relevant co-morbidities that result in hypogonadism, either by association with their kidney disease or as the cause of it. These include obesity, diabetes mellitus—both type 1 and type 2, depression, and hypertension (21, 22). In particular, raised inflammatory biomarkers have been shown to be associated with low testosterone levels (23). Thus, a variety of inflammatory comorbidities are likely to be implicated, together with CKD *per se* in the development of hypogonadism.

### Medications That Influence Sex Hormone Abnormalities in CKD

Prescribed medications may interfere with testosterone production. These include angiotensin-converting enzyme (ACE) inhibitors, angiotensin receptor blockers (ARBs), spironolactone, and corticosteroids (24), which are often prescribed to patients with CKD. However, the case for ACE inhibitors and ARBs is controversial. While one study has argued that suppression of testosterone production is the link between ACE inhibitors and lower hemoglobin levels in hemodialyzed patients (25), another larger epidemiological investigation noted no apparent effect of ACE inhibitor/ARB use on serum testosterone levels in men receiving chronic hemodialysis (23).

In addition to these drugs, other agents sometimes used to treat men with CKD, which can exhibit antiandrogenic properties include cimetidine, ranitidine, and methotrexate.

## ED in CKD

Erectile dysfunction is defined as the persistent inability to achieve or maintain a penile erection sufficient for satisfactory sexual activity. In CKD stage 5-D, the prevalence exceeds 80% (26). Although ED appears to become more common with declining GFR, the rate is high for all kidney disease from CKD stage 3 onward.

### Mechanisms of ED

Multiple mechanisms for the development of ED in CKD have been studied. Table 1 outlines these, and they are discussed individually below.

**Table 1:** **Mechanisms of erectile dysfunction in chronic kidney disease**.

Vascular	Penile atherosclerotic disease
Endocrine	Hypogonadism and hyperprolactinaemia
Neurologic	Uremic and diabetic autonomic neuropathy
Pharmacologic	Histamine antagonists, antihypertensives
Anemia	Oxygen delivery to corpora cavernosa
Hyperparathyroidism	Penile calcification and hyperprolactinaemia

#### Vascular

Chronic kidney disease is itself a risk factor for the development of atherosclerotic vascular disease. Even young patients receiving dialysis therapy have a cardiovascular mortality equivalent to octogenarians in the general population (27). Vascular disease has a close association with ED (28). This is unsurprising, given that the penile circulation is no less vulnerable than the remainder of the vascular tree to endothelial dysfunction and atherosclerosis. Impaired penile blood supply contributes to the development of ED.

Therefore, it might be expected that there will be a correlation between vascular disease and ED in CKD patients and that the excess of vascular disease in this population is partly responsible for the burden of ED. Indeed, an association has been shown between severe ED and coronary artery calcium score in dialysis patients (29) and between ED and carotid intima-media thickness in hemodialysis recipients (30).

In a small study of 20 patients with CKD stage 5-D and ED, the combined use of pharmacocavernosography and pharmacocavernosometry found evidence of cavernosal artery occlusive disease in 78% (31). This supports the contribution of penile atherosclerotic disease to ED in CKD.

#### Endocrine

As outlined above, the hypothalamic–pituitary–gonadal axis is disturbed in CKD. Hypogonadism is relatively common in this population and ED occurs in concert with the other physical consequences of testosterone deficiency.

Peripherally, testosterone affects the majority of pathways involved in normal erectile function, including smooth muscle cell structure, function, and innervation and maintains fibroelasticity of the corpus callosum. There is a central effect of testosterone deficiency *via* reduction in sexual desire, which occupies an important role in ED. It is generally accepted that testosterone is required for the generation of nitric oxide *via* upregulation of neuronal nitric oxide synthase. This nitric oxide production is associated with the increased intracavernous pressure required for erectile function (32, 33).

In addition to its effects *via* hypogonadotropic hypogonadism, prolactin has been suggested to have a direct central effect on sexual arousal that contributes in a testosterone-independent manner to ED (34).

#### Neurologic

Adequate parasympathetic outflow is essential for cavernous sinusoidal relaxation and hence increased cavernosal blood flow. With autonomic neuropathy, the parasympathetic nervous system is impaired and this results in compromised erectile function (32). Diabetes mellitus is now the commonest cause of CKD stage 5-D. Autonomic neuropathy frequently accompanies type 1 diabetes mellitus when there is evidence of end-organ damage elsewhere. It is known that severity of cardiovascular autonomic neuropathy in patients with type 1 diabetes correlates with ED (35).

In addition to diabetes as a potential cause of autonomic neuropathy, it has been shown that uremia *per se* may result in autonomic nervous system dysfunction (36, 37). This neuropathy appears irreversible; certainly, it is unresponsive to dialysis therapy. Significantly, there is a predilection for failure of the parasympathetic arm of the autonomic nervous system. A direct correlation between autonomic neuropathy in CKD and reduced nocturnal penile tumescence has been demonstrated (38). Thus, CKD-induced autonomic neuropathy, often abetted by diabetic autonomic neuropathy, constitutes an additional promoter of ED.

#### Pharmacologic

An extensive pharmacopeia is often found in the management of CKD. Hypertension is not universal, but the control of blood pressure remains the mainstay of therapy in many progressive kidney diseases despite therapeutic and diagnostic advances. In addition, hypertension is widespread in those patients requiring dialysis therapy, particularly in view of the chronic extracellular fluid volume expansion seen in this group. Antihypertensive agents comprise various drug classes, and some of those most frequently prescribed are associated with ED. The prescription of medications for other indications is common in CKD patients, and some of these too can be implicated in ED.

Of the antihypertensive drugs, diuretics, beta blockers, calcium-channel blockers, and methyldopa are most strongly associated with ED. Other prescribed culprit medications include histamine antagonists and digoxin. Antidepressants present an interesting case. While depression is associated with ED and is a frequent comorbidity in CKD patients, treatment is not always associated with an improvement in ED (39). This might be explained by the known propensity of certain antidepressant medications to cause ED, particularly selective serotonin-reuptake inhibitors and tricyclic antidepressants. An alternative explanation might be that conventional antidepressant medication is lacking in efficacy generally in this patient group. It is recognized that there is very little good quality data in this area, but that patients with CKD stage 5-D have not been shown to derive much benefit from antidepressant drugs (40).

#### Anemia

Anemia is approximately twice as common in CKD as in the general population, and the prevalence rises in parallel with falling GFR (41, 42). This surplus prevalence is accounted for by erythropoietin deficiency, which increases in likelihood and severity as kidney function declines. An association between anemia and ED has been reported in CKD (43). In anemia, there is a reduction in the delivery of oxygen to the tissues, and this includes the corpora cavernosa. Decreased oxygen availability is implicated in impaired nitric oxide generation within the erectile tissues and reduced erectile function (43, 44).

In current practice, patients with CKD stages 3–5 are treated with iron and erythropoiesis stimulating agents (ESAs) to maintain the hemoglobin within target parameters. This means that few such patients should be moderately or severely anemic. However, acknowledging an elevated risk of hypertension, stroke, and dialysis fistula thrombosis with increasing ESA dose, a typical target range for hemoglobin is between 10.0 and 11.5 g/dl (45). A consequence of this is that the majority of male CKD stage 4 and 5 patients will be mildly anemic whether treated or not, compared to the normal range for healthy individuals. It is conceivable this will have an impact on ED, in conjunction with other risk factors.

#### Hyperparathyroidism

Varying degrees of secondary hyperparathyroidism, in some cases progressing to tertiary hyperparathyroidism, are near-ubiquitous in CKD stages 4–5. It has been suggested that hyperparathyroidism might contribute to the development of ED, whether independently or perhaps *via* prolactin release. Supporting evidence has come from the observation that penile calcification in a small group of hemodialysis patients correlated with ED (46). An observational study in which parathyroid hormone (PTH) was partially suppressed with calcitriol found an improvement in plasma testosterone levels and reported sexual function (47). Most intriguingly, a series of 20 hemodialysis patients in Taiwan that underwent subtotal parathyroidectomy for hyperparathyroidism was found to have improved sexual function 3 months postoperatively. This was associated with unaltered testosterone levels but a reduction in PTH and prolactin (48).

### Relationship of ED to GFR

Given that CKD is a powerful risk factor for ED, it is intuitive that the severity of CKD will determine the degree of risk for the development of ED. Many of the etiological factors in CKD-associated ED will progress as GFR falls. These include hyperparathyroidism, hypogonadism, autonomic neuropathy, and vascular disease. Affirming this reasoning with data from the literature is more difficult due to heterogeneity in study populations, small sample sizes, and variation in study tools used to identify ED (49). An underpowered Brazilian study found a trend toward increasing prevalence of ED with advancing stages of CKD (50). In a series of non-dialyzed patients, rates of ED were 72.3% with CKD stage 3, 81.5% with CKD stage 4, and 85.7% with CKD stage 5-ND. However, this apparent progression did not reach statistical significance. In a systematic review and meta-analysis of 50 studies, the overall rate of ED in CKD 5-D patients was 75% with a lower rate of 59% in recipients of kidney transplants (CKD 5-T) (49). This might suggest that the restoration of GFR provided by the transplanted kidney results in improved erectile function.

A study examining the risk of ED following radical versus partial nephrectomy found a significantly higher rate of *de novo* ED in those subjected to radical surgery (51). This suggests that a greater reduction in nephron mass resulted in a greater risk of ED development.

### Risk Factors for ED in CKD

Risk factors for any degree of ED in dialysis patients are numerous, and perhaps a degree of caution needs to be exercised in their interpretation given the high prevalence of reported symptoms. Nevertheless, they include symptoms of depression, low educational attainment, diabetes, cardiovascular disease, hypertension as the stated cause of CKD, lower delivered dialysis dose, and alcohol abuse (39). Previous kidney transplantation and wait-listing for transplant appear to be protective. In a large meta-analysis of CKD patients with stage 4, 5-ND, 5-D, and 5-T disease, the strongest correlates for development of ED were diabetes (principally type 2 diabetes mellitus), depression, and increasing age (49).

### Psychological Factors

Historically, ED was attributed primarily to psychological causes. In more recent times, the varied organic disorders that make up the majority of the burden of disease in ED have been given increasing prominence. Disentangling cause from effect where there is psychological morbidity and ED can be difficult. Nevertheless, psychological screening is essential in considering the etiology of ED.

Symptoms of depression have been strongly associated with the development of ED in patients with CKD (39), so too has unemployment. In CKD stage 5-D, depression may follow the loss of a primary role in occupation or the family or reflect reduced physical or cognitive function (52). In some dialysis patients, a sense of helplessness prevails, due to fears of complications or hospitalization.

Overall, 20–30% of dialysis patients have been shown to suffer from clinical depression (53, 54). A study of CKD stage 4–5 subjects with a GFR below 20 ml/min found that 25% had major depression and a further 20% had minor depression as measured by a structured interview questionnaire (55).

### Effect of Renal Transplantation on ED

It has been shown that renal transplantation can reverse the endocrine abnormalities associated with CKD stage 5 in the majority of allograft recipients. The restoration of kidney function, at least to a degree, improves prolactin clearance, and thus, LH and testosterone levels normalize (56, 57). Hyperparathyroidism and anemia also improve after successful transplantation. Vascular disease persists, however, although its progression might be retarded. A need for antihypertensive drugs or diuretics continues for some. Hence, a reduction in ED severity and prevalence might be expected posttransplant, but this will depend upon the predominant etiological mechanism. It is also worth observing that the majority of transplant recipients have spent time as dialysis patients and that their dialysis vintage is likely to influence erectile function after transplantation, as it does other outcomes.

The published data supports this reasoning. ED is still widely prevalent in kidney transplant recipients, but not to the same degree as in CKD stage 5-D. If the prevalence of ED in dialysis patients is taken to be around 80% (26, 57), then epidemiological results suggest a lower level of approximately 30–50% following transplantation (57–59). Even allowing for the younger age and lower comorbidity of the transplanted population relative to dialyzed patients, this indicates that a sizeable proportion of men see improved erectile function following engraftment.

The availability of published data to compare erectile function pre- and post-transplantation is limited. A Chinese transplant center used the International Index of Erectile Function (IIEF) scoring system in 24 patients with CKD stage 5-D before and after a kidney transplant. In all, 87.5% experienced some degree of ED before surgery and 46% afterward (57). The majority of 15 Iranian recipients of living donor organs reported better erectile function after kidney transplantation (60). Forty-seven percent had ED prior to surgery, perhaps a low figure. A higher rate of posttransplant ED was found in a larger cross-sectional study, reaching 55.7% of 271 recipients (59). Here, time on dialysis prior to transplantation was associated with negative effects upon erectile function in the posttransplant period. Interestingly, a single small study examining ED after simultaneous kidney-pancreas (SKP) transplantation concluded that successful combined transplantation restored near-normal levels of erectile function (61). Whether this simply reflects the lower age and dialysis vintage of the typical SKP recipient is unclear.

### Quality of Life Effects of ED in CKD

Unreported, unrecognized, or untreated ED has substantial implications for the sufferer. In a historical study of 17 married couples where one partner was treated with dialysis, marital discord and sexual dysfunction were closely linked (62). Quality of life, self-esteem, anxiety, and depression have been shown to be negatively influenced by ED in patients with CKD (63, 64). An inability to achieve satisfactory intercourse has adverse effects on quality of life in particular. According to one survey, the majority of peritoneal dialysis patients did not have intercourse, although 50% of those surveyed would wish to (55). It is worth observing that quality of life and ED are associated in both directions: impaired quality of life due to kidney disease can worsen ED.

## Impact of CKD on Libido

Given the high prevalence of hypogonadism and ED in men with CKD, it is not unexpected that diminished libido is also a feature of the disease. One qualitative study using Diagnostic and Statistical Manual of Mental Disorders (DSM-III-R) criteria found that the majority (57–60%) of enrolled dialysis patients suffered “Hypoactive Sexual Desire Disorder” (65). This was compared to control groups with functioning kidney transplants or rheumatoid arthritis and no kidney disease, in whom the frequency was less than 13%, and the difference was statistically significant. It is established that libido is decreased in men with testosterone deficiency (66). Hypogonadism is likely to be an important factor in the reduced libido commonly seen in patients with CKD, particularly in CKD stage 5-D. In addition to the pathophysiological effects of endocrine dysfunction, CKD patients experience diminished quality of life due to impaired health (67), and this will also contribute to a reduction in libido. It has been shown that erythropoietin treatment of anemia in CKD results in improved libido, indicating an impact of low hemoglobin levels in reducing libido (68).

## Body Image and CKD

A significant proportion of adult CKD patients, particularly those with CKD stage 5, developed their kidney disease as children or adolescents. As a consequence, normal physical development is impaired despite ideal management. In addition to short stature, the body in patients with CKD stage 5-D or 5-T is altered both by the effects of kidney disease and the impact of treatment. There may be scars from vascular access procedures, peritoneal access surgery, or kidney transplantation. Hemodialysis or peritoneal dialysis catheters protrude from the skin, arteriovenous fistulae in the upper limbs are visible if the arms are exposed and the abdomen may distend from peritoneal dialyzate or enlarged polycystic organs. The eventual loss of native renal function and cessation of urine output also influence body image adversely.

Changing modality of renal replacement therapy from dialysis to kidney transplantation has an impact on body shape. Weight gain after kidney transplantation is common and can result in obesity in patients with a previously normal body mass index (69). Immunosuppressive medication prescribed after transplantation can also alter physical appearance, particularly where hirsutism develops with ciclosporin treatment (70).

A United Kingdom study of young adults aged 16–30 years with CKD stages 5-D and 5-T found that the impact of kidney disease and its treatment on body image had exerted a profound effect on intimate relationships (71). Using a bespoke, internally validated questionnaire, an Iranian study of 42 hemodialysis and 42 kidney transplant patients found that both groups had altered body image, with a greater disturbance in the body image of the hemodialysis patients that reached statistical significance (72).

## Effect of CKD on Male Fertility

It is well established that men with CKD stage 5-D have significantly lower fertility than members of the general population (56, 73, 74). In one small study of hemodialysis patients, more than 40% had oligospermia or azoospermia (56). This occurs due to deficiencies in sperm morphology, motility, and density (75). A recent Scandinavian study found a correlation between higher stages of CKD and declining semen quality (76). Testicular volume is reduced in dialysis patients in comparison to normal controls (77). The exact mechanism for impaired spermatogenesis remains unclear. The endocrine derangements described earlier go some way to accounting for abnormal sperm production. The presence of oxidative stress has also been identified as a potential contributor to testicular dysfunction (77, 78). Oxidative stress is induced by various inflammatory pathways in CKD and exacerbated by the use of glucose-based peritoneal dialysis solutions or the extracorporeal circuit in hemodialysis. A single study has suggested that reduced expression of the cystic fibrosis transmembrane conductance regulator by spermatozoa is associated with impaired sperm quality in uremia (79). However, the exact role of this protein in fertility is not established.

There are other contributory factors that may apply in a proportion of men with CKD. In patients with adult polycystic kidney disease, cyst development is not confined to the kidneys. Seminal vesicles can become cystic, and this may be an additional influence upon testicular function (80). Some patients with systemic vasculitis, lupus nephritis, or primary glomerular disease are treated with cytotoxic medication such as chlorambucil or cyclophosphamide. These drugs possess gonadal toxicity (81), and if such patients progress to CKD stage 5-D, there will be an additional risk factor for infertility.

## Management of Sexual Dysfunction in CKD

### Erectile Dysfunction

A number of interventions have been proposed for the treatment of ED in CKD patients. However, a recent systematic review concluded that there is a lack of safety data for any therapeutic options for ED in this population (82). Small clinical trials constitute the entirety of the evidence for efficacy even for the most comprehensively studied interventions. For most of the plausible treatment strategies, there is no published clinical trial data at all.

The initiation of dialysis therapy for patients with CKD stage 5 improves some clinical characteristics but has not been shown to improve ED (83). The dialysis modality is probably unimportant. While the reported rate of ED in peritoneal dialysis patients might be lower (84), these patients are often younger than their hemodialyzed counterparts and have a lower comorbidity burden. Conversely, a number of studies have found that kidney transplantation has a beneficial effect upon sexual function in a significant proportion of patients previously treated with dialysis. Longitudinal follow-up of hemodialysis patients who then received a kidney transplant has found a posttransplant improvement in the IIEF score (57, 83, 85). Other small studies seeking to compare erectile function between groups of dialyzed and transplanted patients have come to similar conclusions, although this study design is less robust (86, 87).

The most comprehensively investigated treatment option for ED in CKD patients is the administration of phosphodiesterase-5 inhibitors (PDE5i). PDE5i have been approved by the US FDA for treatment of ED since 1998. Three randomized controlled trials in CKD have compared PDE5i with placebo and demonstrated a consistent improvement in IIEF scores (88–90). Two of these studies recruited only kidney transplant recipients, the third investigated PDE5i in hemodialysis patients. The medications used were sildenafil in two trials and vardenafil in the other. In addition to this single randomized controlled trial of sildenafil in hemodialysis patients, three small uncontrolled studies and a randomized crossover trial of PDE5i have been published in dialysis (91–94). Table 2 summarizes the published data from dialysis patients including the single double-blind, randomized controlled trial. The intervention groups in all studies of PDE5i in transplanted or dialyzed subjects were small, ranging from 20 to 39 patients (88–94). A recent Cochrane Review concluded that there was modest evidence for the efficacy of PDE5i in CKD, recognizing the paucity of well-designed randomized controlled trials and the small sample sizes (82).

**Table 2:** **Studies of phosphodiesterase-5 inhibitors in dialysis patients**.

Study	Design	Population	Drug	Outcome
Türk et al. (91)	Intervention; no control group	35 HD patients, 15 PD patients	Sildenafil	Significant improvement in International Index of Erectile Function (IIEF) score
Chen et al. (92)	Intervention; no control group	34 HD patients, 1 PD patient	Sildenafil	Effective in 80% of patients (IIEF)
Yeniçerioğlu et al. (93)	Intervention; no control group	30 HD patients, 11 PD patients	Sildenafil	IIEF score: 71% recovered erectile function
Seibel et al. (90)	Double-blind, randomized, placebo-controlled	41 HD patients	Sildenafil	IIEF score: 85% improvement with sildenafil versus 9.5% placebo
Turk et al. (94)	Randomized crossover	32 HD patients	Sildenafil and Vardenafil	IIEF scores significantly improved by both drugs

A second therapeutic intervention that has attracted some attention historically is zinc supplementation. This has been studied as an oral supplement or as an additive to dialyzate in hemodialysis. Dialyzate zinc administration yielded no benefit in comparison to placebo when testosterone or gonadotropin levels were measured (95, 96). In contrast, a small double-blind, placebo-controlled trial of oral zinc in hemodialysis patients demonstrated an improvement in endocrine parameters (increased testosterone, reduced LH) along with an increase in potency and frequency of intercourse in the zinc-treated subjects (97). It is worth noting that zinc studies are something of a historical artifact, with no major contribution to the literature in the last 30 years.

Having recognized the significant prevalence of hyperprolactinemia in the CKD stage 5-D population, bromocriptine has been investigated in the past as a treatment option in view of its ability to suppress prolactin levels. A small, uncontrolled interventional study found a normalization of prolactin levels in seven hemodialysis patients was associated with an improvement in self-reported sexual function and testosterone levels (98). Two slightly larger randomized crossover studies reported a high rate of intolerance of bromocriptine but a subjective improvement in ED when compared to placebo (99, 100). These accumulated data seem insufficient to endorse bromocriptine as a valuable intervention for ED in the CKD setting.

Testosterone replacement has also been considered as a means of addressing CKD-associated endocrine dysfunction and subsequent ED. It has been observed that men suffer a more rapid progression of renal disease than women (101). Laboratory studies in animals suggest that testosterone might bear some responsibility for this, although corroborating clinical data in humans are lacking. Nevertheless, the use of testosterone supplementation in patients with pre-dialysis CKD might be regarded with a degree of caution. This concern is of less relevance in established CKD stage 5-D. Setting this aside, there is also an understandable reluctance to prescribe testosterone replacement through anxiety that prostate cancer might be promoted. Such fears have not been comprehensively supported by the literature. No statistically elevated risk of prostate cancer was found in middle-aged and older men treated with testosterone replacement (102). Notwithstanding this, prostate specific antigen (PSA) monitoring is recommended by the Endocrine Society for those men receiving testosterone therapy for androgen deficiency (103). Salt and water retention has been reported in hemodialysis patients treated with testosterone, and its use in CKD patients is considered a caution.

The recognition that low testosterone levels are associated with increased cardiovascular mortality has given further impetus to pharmacological testosterone replacement in patients with and without CKD. Unfortunately, it still remains unclear whether such replacement actually worsens cardiovascular and all-cause death in treated patients (104).

Despite these reasons for circumspection and insufficient data to assure long-term safety, a case can be made for the replenishment of testosterone to physiological levels in androgen-deficient men (24, 103). Therefore, this will apply to a sizeable proportion (40–60%) of CKD patients, as described above. Contra-indications include the presence of prostate cancer or palpable prostatic nodules, PSA above 4 ng/ml, untreated severe obstructive sleep apnea, and prostatic hypertrophy causing severe lower urinary tract symptoms. Benefits of correcting hypogonadism might include improvements in ED, as evidenced by two small uncontrolled case series (105, 106), although a third study found little gain from treatment (107).

In cases where the administration of certain medications might be implicated in the development of ED in CKD patients, a careful review of treatment indications and alternative interventions is warranted. A reappraisal of prescribed antihypertensive treatment can highlight drugs most likely to promote or induce ED. Thiazide diuretics and beta blockers in particular are strongly associated with ED (32). Alternative agents such as ACE inhibitors or ARBs (despite their potential effects on testosterone) can be offered, and alpha blockers are usually less problematic in this situation. Histamine antagonists such as ranitidine are also likely culprit medications and might be exchanged with proton pump inhibitors. A history of high alcohol intake, while less common in patients receiving dialysis therapy, should prompt lifestyle advice and the offer of behavioral intervention.

Surgical treatment remains an option in CKD patients with sexual dysfunction. The only non-pharmacological therapy to have been investigated and published in this context is vacuum detumescence therapy, which showed promising results in a small cohort of 26 dialysis patients (108). It is generally considered that the use of penile prostheses is justifiable if pharmacological therapy has failed. In the dialysis patient, this might be deferred until after transplantation where reasonable, given the expectation that a significant proportion will regain erectile function posttransplant.

Acknowledging the connection between depression, quality of life and sexual dysfunction in CKD—particularly CKD stage 5—other interventions might prove productive. Antidepressant pharmacotherapy has a limited and conflicting evidence base, but psychosocial intervention with cognitive behavioral therapy (CBT) has also been studied. A randomized crossover trial in New York recruited hemodialysis subjects and compared immediate CBT with a wait-listed group. The CBT was delivered chairside during dialysis sessions. Treated patients fared better than those as yet untreated in various domains including depression scores and quality of life (109). A randomized controlled trial of CBT versus no intervention in an Indian hemodialysis population found significantly improved anxiety and depression scores in the CBT group (110).

### Infertility

Specific treatment of male infertility in CKD is poorly studied, and there is a paucity of published work. It is recognized that successful kidney transplantation results in improved endocrine parameters and also higher semen quality. Sperm motility, morphology, and number are increased, although this may take some time after engraftment of a functioning kidney (74, 108).

## Conclusion

The male endocrine system is substantially deranged with progressive CKD. A major consequence is a reduction in effective testosterone, which arises due to both primary and hypogonadotrophic hypogonadism as well as altered SHBG levels. Although low testosterone is associated with both morbidity and mortality, the influence of increasing patient age is difficult to separate from direct effects of androgen deficiency. Testosterone replacement for androgen-deficient male CKD patients has some support from expert guidelines, but lacks a robust evidence base and carries theoretical risks.

In addition to endocrine dysfunction, the intimate relationships of CKD patients are challenged by altered body image, diminished libido, and reduced fertility.

Management of ED in CKD centers upon the prescription of phosphodiesterase inhibitors. Pragmatic review of antihypertensive therapy can help to minimize ED as a side effect of certain medications.

## Author Contributions

The author performed the literature searches, reviewed the papers and wrote the text.

## Conflict of Interest Statement

The author declares that the research was conducted in the absence of any commercial or financial relationships that could be construed as a potential conflict of interest.
